# AdaptMol: domain adaptation for molecular image recognition with limited supervision

**DOI:** 10.1186/s13321-026-01209-2

**Published:** 2026-05-02

**Authors:** Feng Hu, Estrid He, Karin Verspoor

**Affiliations:** School of Computing Technologies, RMIT University, Melbourne, VIC Australia

**Keywords:** OCSR, Deep learning, Transformer, Domain adaptation, Unsupervised learning

## Abstract

Optical Chemical Structure Recognition (OCSR) aims to convert two-dimensional molecular images into machine-readable formats such as SMILES strings. Deep learning has substantially improved OCSR performance, yet most methods rely on synthetic training data and struggle to generalize to real-world inputs, especially hand-drawn diagrams, where stroke width, geometry, and drawing conventions vary widely across individuals. In this work, we propose an image-to-graph model AdaptMol that enables effective transfer from synthetic to real-world data without requiring manual graph annotations in the target domains. AdaptMol is an integrated pipeline that starts with training a base model on synthetic data, and then refines model representations through unsupervised domain adaptation and self-training. Our key insight is that bond features are domain-invariant in nature; they encode structural relationships between atoms that are independent of visual variations across domains. Thus, during domain adaptation, we align bond-level feature distributions via class-conditional Maximum Mean Discrepancy (MMD) to enforce cross-domain consistency. We also design a comprehensive data augmentation strategy to enhance the robustness of the base model, facilitating stable self-training on unlabeled target samples. On hand-drawn molecular images, our model achieves 82.6% accuracy and outperforms the best prior method by 10.7 points, while maintaining competitive performance across four benchmarks comprising molecular images from scientific literature and patent documents.

**Scientific contribution**

We propose AdaptMol, an image-to-graph model that predicts molecular structures as graphs of atoms and bonds, achieving effective transfer from synthetic to hand-drawn molecular images without requiring target domain graph annotations. We combine class-conditional Maximum Mean Discrepancy to align bond features across domains with comprehensive data augmentation to increase training data variation, jointly improving base model accuracy sufficiently for self-training and addressing the critical failure mode of prior approaches that begin with insufficient accuracy. We further introduce a dual position representation that supervises atom positions through both discrete coordinate tokens and continuous spatial heatmaps to reduce false positives in atom localization.

## Introduction

Optical Chemical Structure Recognition (OCSR) is the task of automatically converting two-dimensional molecular images into machine-readable representations such as Simplified Molecular Input Line Entry System (SMILES) [1] or Molfile. Millions of molecular structures in scientific publications and patent documents exist only as images and remain inaccessible to computational analysis [2], and OCSR provides a way to extract this information automatically. This capability is essential for data-driven approaches in drug discovery [3, 4], materials design [5, 6], and synthesis planning [7, 8], all of which depend on large-scale chemical datasets to train machine learning models.

Despite its importance, OCSR remains a challenging problem. Molecular depictions vary considerably across publications, with differences in atomic label fonts, bond drawing styles, and overall graphical conventions, making it difficult for models to remain robust across diverse sources. In addition, molecular structures themselves exhibit vast combinatorial diversity, as countless combinations of atoms and functional groups create an exponentially expanding chemical space that models must learn to generalize across. Finally, the task demands exceptionally high precision, since even a single error in identifying an atom or bond can fundamentally alter the resulting structure and render it unusable for downstream applications.

To address these challenges, early OCSR approaches relied on rule-based pipelines involving image preprocessing, detection, OCR, and heuristic assembly, requiring extensive manual tuning [9–11]. Recently deep learning based methods have attracted extensive attention, as they can learn statistical rules and patterns from training data. These can be divided into two paradigms. Image captioning methods formulate the OCSR as an image-to-sequence task, directly generating machine-readable SMILES strings from molecular images via encoder-decoder architectures. An early approach proposed by Staker et al. [12] employed Convolutional Neural Network (CNN) encoders [13] with Recurrent Neural Network (RNN) decoders [14], and subsequent works mainly focus on refining these through more advanced architectures such as Inception [15] or SwinTransformer [16] encoders and LSTM [17] or Transformer [18, 19] decoders. Graph reconstruction methods generate an intermediate molecular graph representation, where atoms or functional groups are nodes and bonds are edges. This graph is then deterministically converted into SMILES or Molfile formats. These methods can be further divided into two categories: two-stage approaches first detect atom and bond positions, then classify their types before assembly [20–22]; end-to-end approaches jointly predict atoms (with coordinates) and bonds through autoregressive sequence generation using encoder-decoder architectures [23, 24]. These task-specific approaches develop deep learning models with architectures and training objectives explicitly designed for molecular structure recognition. Recently, an alternative line of work has explored applying large language models (LLMs), which are significantly larger foundation models with broad reasoning capabilities, to OCSR. However, as demonstrated in ChemVLM [25], both general-purpose and chemistry-specialized multi-modal LLMs significantly underperform task-specific methods, underscoring the continued need for specialized architectures in molecular structure recognition.

We focus on task-specific graph reconstruction approaches for OCSR. Graph reconstruction methods address the syntax brittleness of image captioning by generating intermediate molecular graphs rather than SMILES strings directly. This eliminates the need to learn complex syntax rules, avoiding character-level errors that invalidate outputs [26, 27]. Additionally, the structured output of atoms and bonds is more constrained than arbitrary SMILES sequences, requiring less training data and making learning more tractable [20]. However, graph reconstruction faces a critical limitation: it requires atom coordinates and bond annotations that are readily available in synthetic datasets but prohibitively expensive to obtain for real-world molecular images. This annotation bottleneck prevents existing methods from generalizing to diverse real-world domains such as hand-drawn molecules and patent documents.

**Fig. 1:**
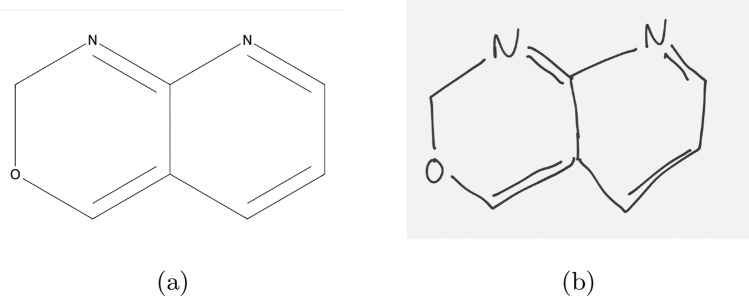
Comparison between **a** synthetic and **b** real hand-drawn molecular images of the same molecular structure. Synthetic samples follow strict drawing rules with uniform fonts and bonds, whereas hand-drawn ones show irregularity and variation typical of human sketches

**Fig. 2:**
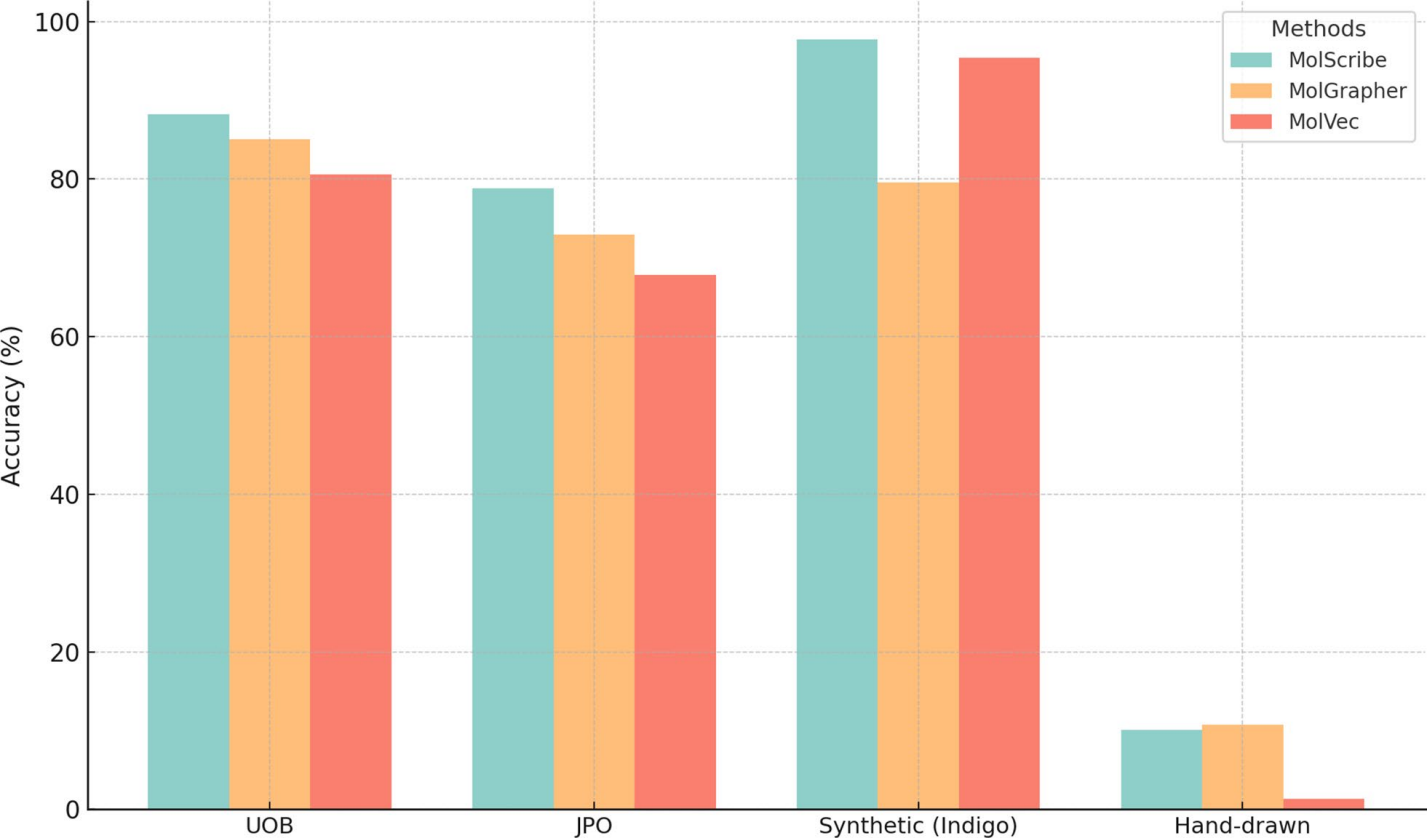
Previous methods (MolScribe [23], MolGrapher [20], MolVec [10]) achieve over 65% accuracy on challenging literature datasets (UOB, JPO) and over 75% on synthetic data (Indigo), but drop below 15% on hand-drawn images, demonstrating the severity of distribution shift between training data and real-world hand-drawn depictions

This annotation bottleneck is compounded by a fundamental distribution gap in existing work: most methods are trained on synthetic images and evaluated on high-quality literature figures, both exhibiting relatively uniform depiction styles. This overlooks the substantial diversity present in real-world molecular documentation, particularly hand-drawn structures appearing in laboratory notebooks, educational materials, and historical chemical literature. As shown in Fig. 1, hand-drawn depictions exhibit significant distribution shifts from synthetic training data: irregular bond lengths and angles, variable stroke widths and thicknesses, inconsistent atomic symbols, and sometimes ambiguous structural features. These combined challenges of lacking annotations and large domain shifts lead to severe performance degradation on hand-drawn molecules, as illustrated in Fig. 2.

Adaptation to hand-drawn images requires learning representations that capture this variability under limited supervision. For image captioning methods, DECIMER-Handdraw [28] addresses this through massive-scale simulation via specialized rendering tools, generating over 38 million synthetic hand-drawn images. However, despite this enormous amount of data, manually designed simulation strategies cannot fully capture the authentic irregularities present in real hand-drawn molecules, limiting the effectiveness of this approach. For graph reconstruction methods, the annotation requirement poses additional challenges, as no rendering tools exist that can simultaneously simulate hand-drawn styles while providing precise atom coordinates and bond types needed for supervision. Rather than developing such complex rendering tools, AtomLenz [29] employs self-training to guide a model to refine its representations with unlabeled data. It employs an exhaustive graph correction pipeline: predicting molecular graphs on unannotated images, then iteratively modifying atoms and bonds until matching valid SMILES strings are found. These refined molecular graphs are used as pseudo-labels for model self-training. However, this iterative graph correction process is time-consuming. It is also ineffective due to the limited accuracy of the base model, which generates unreliable pseudo-labels.

The limitations of previous works motivate our approach: we leverage the structured advantages of graph reconstruction while addressing its annotation requirements through efficient domain adaptation to target domains where only SMILES strings are available. We propose AdaptMol, which formulates OCSR as molecular graph generation with a domain adaptation pipeline combining unsupervised alignment and self-training. Specifically, AdaptMol  operates in three stages. First, we train an end-to-end graph reconstruction model on synthetic data with comprehensive data augmentation, incorporating our novel dual position representation that supervises atom positions through both discrete coordinate tokens and continuous spatial heatmaps for better atom localization. Second, we apply a class-conditional Maximum Mean Discrepancy (MMD) [30] criterion to align bond-level feature distributions between synthetic and target domain data. Third, we perform self-training using model predictions on unlabeled target images that can be validated against reference SMILES strings. Our key insight is to prioritize base model accuracy through MMD alignment and data augmentation before self-training, rather than relying on exhaustive post-hoc correction of invalid predictions. This enables effective pseudo-label generation without additional correction steps. We focus on hand-drawn molecular images as our target domain, representing a challenging scenario with extreme distribution shift from synthetic training data.

Extensive experiments demonstrate that our method achieves effective cross-domain transfer without requiring massive simulation data or target domain graph annotations. On hand-drawn molecular images, we achieve 82.6% accuracy, outperforming both image captioning and graph reconstruction baselines, while ranking first across four standard benchmarks and achieving the highest atom localization accuracy among all compared methods. The main contributions are as follows: (1) We propose a data-efficient domain adaptation pipeline combining comprehensive augmentation, bond-level MMD alignment, and self-training for annotation-free transfer, achieving superior performance; (2) We design a dual position representation combining coordinates and heatmaps that reduces false positives and improves atom localization; (3) We achieve the best reported accuracy on hand-drawn molecular recognition while requiring significantly less training data than prior methods.

## Methods

### Task definition

We formalize molecular image recognition as a graph generation task. Given an input image $I \in \mathbb {R}^{H \times W \times 3}$, the goal is to generate a molecular graph $G = (N, E)$, where $N=\{n_1,n_2,\dots ,n_n\}$ denotes the set of atoms in the molecule, and each atom $n_i=(l_i, x_i, y_i)$ is represented by a label $l_i$ together with its two-dimensional coordinates $(x_i,y_i)$ in the image *I*. The set *E* represents all chemical bonds in the molecule and can be expressed as an $n \times n$ adjacency matrix $E=[b_{ij}]_{n\times n}$, where *n* is the number of atoms and $b_{ij}$ encodes the bond type between atoms *i* and *j* (single, double, triple, aromatic, solid, wedges, or none). The generated graph is then deterministically converted to SMILES or Molfile format for evaluation.

### Model architecture

**Fig. 3:**
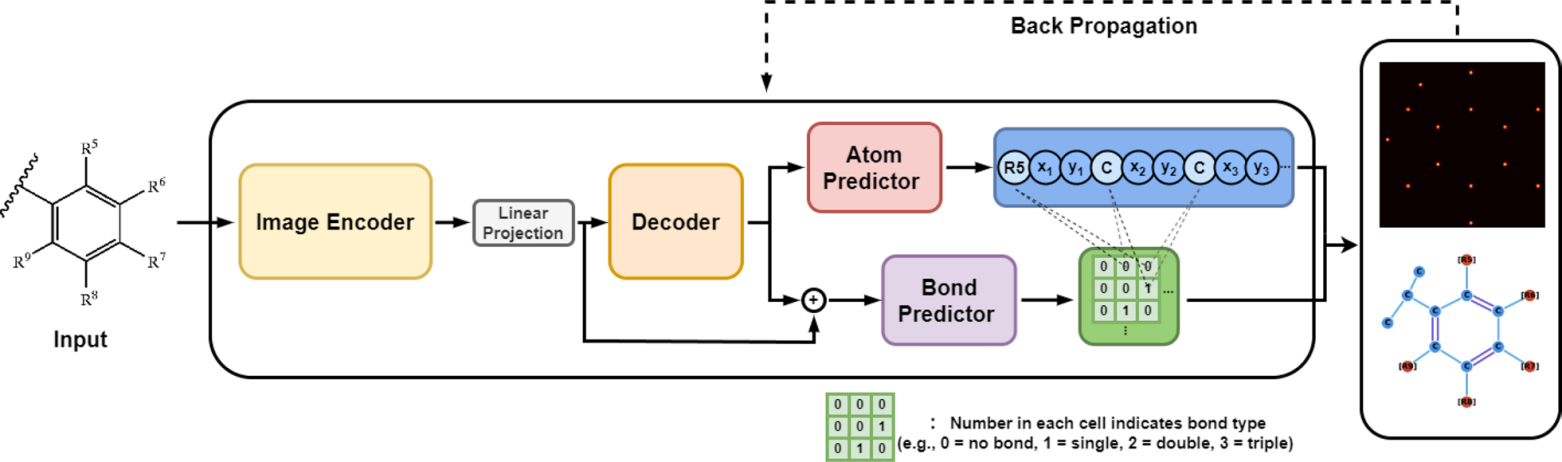
Overview of our encoder-decoder framework for molecular graph generation. The encoder extracts visual features from input images, which are processed by a decoder to generate atom and bond predictions through specialized prediction heads

Our model follows a similar architecture to MolScribe [23], employing a joint learning framework that combines atom detection and bond classification. For atom detection, following Pix2Seq [31], the atom predictor treats detection as sequence generation, autoregressively producing a sequence of atoms with their spatial locations and element types. For bond classification, the bond predictor classifies bond types between each atom pair. Figure 3 illustrates our encoder-decoder framework. The encoder extracts visual features from the input image, which are projected to the decoder’s hidden dimension. The decoder autoregressively generates a sequence of hidden states, which are consumed by both the atom predictor and the bond predictor.

#### Atom sequence generation

The atom predictor projects decoder outputs to vocabulary logits $\textbf{Z} \in \mathbb {R}^{B \times N \times V}$, where *B* is the batch size, *N* is the sequence length, and *V* is the vocabulary size containing atom element types and discretized coordinate bin tokens. Tokens are selected greedily at each step (argmax over softmax probabilities), producing a sequence of atoms with their spatial locations:

1 $$\begin{aligned} S_N = [l_1, x_1, y_1, l_2, x_2, y_2, \dots , l_n, x_n, y_n], \end{aligned}$$

where $l_i$ is the label of the *i*-th atom and $(x_i, y_i)$ are the discrete coordinate bin indices.

Sequential token generation provides sparse supervision for each coordinate independently, which can lead to spatial inconsistencies and false positive predictions. To address this, we introduce dual position representation by additionally predicting a 2D spatial heatmap $\textbf{H} \in \mathbb {R}^{h \times w}$. Specifically, given sequence logits $\textbf{Z}$, we apply softmax and extract x-coordinate and y-coordinate probability distributions $P_x, P_y$ by indexing into the coordinate token positions in the vocabulary. We construct the heatmap by aggregating the joint spatial distributions of all atoms:

2 $$\begin{aligned} \textbf{H} = \text {Upsample}\left( \sum _{i=1}^{n} P_y^{(i)} \otimes P_x^{(i)}\right) \end{aligned}$$

where each outer product $P_y^{(i)} \otimes P_x^{(i)}$ creates a 2D probability density centered at atom *i*’s predicted location, and the summation aggregates all atom positions into a unified spatial density map, which is then upsampled to the target resolution ($h \times w$) via bilinear interpolation. This heatmap exhibits bright spots at atom locations and dark backgrounds elsewhere. During training, this heatmap is supervised with Gaussian distributions centered at ground-truth atom positions, providing dense spatial guidance that complements discrete token predictions, thereby ensuring coordinate consistency and mitigating spurious outputs.

#### Bond classification

The bond predictor generates the adjacency matrix $E \in \mathbb {R}^{n \times n}$ encoding bonds between *n* atoms. Given the decoder hidden states $\textbf{X} \in \mathbb {R}^{B \times T \times D}$ and the predicted atom sequence $S_N$ (Equation 1), we extract atom-level representations by identifying the position of each atom’s y-coordinate token in the sequence. Since each atom is represented by three consecutive tokens $(l_i, x_i, y_i)$, the y-coordinate position contains the most comprehensive information about the atom due to the autoregressive nature of the decoder. For each atom *i*, we extract the corresponding hidden state at position $y_i$ from $\textbf{X}$, yielding atom features $\textbf{F}_{\text {atom}} \in \mathbb {R}^{B \times n \times D}$. To enrich these features with visual context, we apply multi-head attention where atom features serve as queries and encoder outputs serve as keys and values. We employ a learnable weighted residual connection to balance the contribution of original atom information and attended visual features:

3 $$\begin{aligned} \textbf{F}_{\text {enriched}} = \text {LayerNorm}(\textbf{F}_{\text {atom}} + \alpha \cdot \text {MHA}(\textbf{F}_{\text {atom}}, \textbf{E}_{\text {vis}})) \end{aligned}$$

where $\textbf{E}_{\text {vis}}$ denotes encoder visual features, $\alpha$ is a learnable scalar weight, and MHA denotes multi-head attention. Finally, the enriched features are passed through a feed-forward network to predict bond types between all atom pairs, producing the adjacency matrix. The intermediate enriched features are used for domain adaptation (Section Bond-level feature alignment via MMD), as they encode domain-invariant structural relationships independent of visual style.

#### Training

The model is trained with a joint learning objective combining three losses. For atom sequence generation, we use label-smoothed cross-entropy loss comparing predicted token sequences against ground-truth sequences $S_{\text {gt}}$ to mitigate overconfidence. For bond classification, we employ weighted cross-entropy loss comparing predicted adjacency matrix against ground-truth bond matrix $E_{\text {gt}}$ to address class imbalance across bond types. Additionally, we introduce a heatmap reconstruction loss using weighted mean squared error between predicted spatial density $\textbf{H}_{\text {pred}}$ (from coordinate probability distributions) and ground-truth heatmap $\textbf{H}_{\text {gt}}$ (Gaussian kernels at annotated atom positions), with higher weights near ground-truth atoms. The total loss is formulated as:

4 $$\begin{aligned} L_{\text {total}} = L_{\text {atom}} + L_{\text {bond}} + \lambda L_{\text {heatmap}} \end{aligned}$$

where $\lambda$ is set to 0.5. Our model requires full supervision including atom coordinates, element types, and bond classifications. However, such detailed graph annotations are unavailable for real-world molecular images. Therefore, all ground-truth labels are obtained from synthetically generated data, as described in the following section.

### Dataset and augmentation strategies

We generally follow existing works to generate synthetic data for training, but we introduce more comprehensive augmentation strategies. Specifically, we construct a synthetic training dataset using RDKit [32] to generate paired molecular images and graph annotations from SMILES strings. For each SMILES string, we first generate a Molfile using RDKit’s 2D coordinate generation, from which we extract atom labels, 2D coordinates, and bond adjacency matrix. Importantly, we sort atoms by their coordinates (top-to-bottom, left-to-right) to establish a consistent ordering, differing from prior work that uses default atom indexing. To increase structural and visual diversity, we design a comprehensive augmentation at both the structural and visual levels during dataset construction.

#### Structure-rendering augmentation

**Fig. 4:**
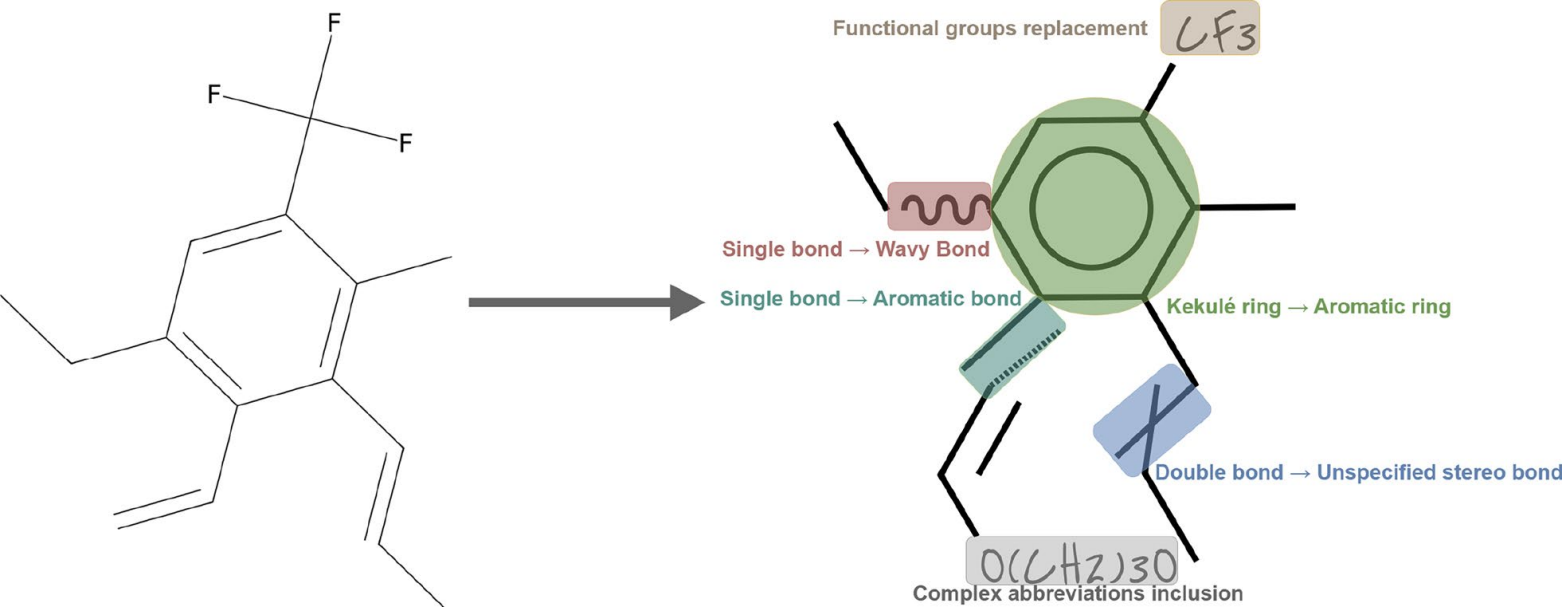
Illustration of structure and rendering augmentations. Structure augmentation includes six main operations: (1) functional group replacement, (2) single-to-wavy bond conversion, (3) single-to-aromatic bond conversion, (4) double-to-unspecified stereo bond conversion, (5) Kekulé-to-aromatic ring conversion, and (6) addition of atoms such as R-groups, functional groups, and complex abbreviations. Rendering augmentation modifies font type/size, bond offset and thickness, and molecular layout

We apply structural modifications and rendering variations jointly during molecule generation to simulate the diversity found in chemical literature. For structural augmentation, we define a set of abbreviation mappings from SMILES substructures to abbreviated labels (e.g., “C” $\rightarrow$ “Me”, “CCC” $\rightarrow$ “Pr”, “c1ccccc1” $\rightarrow$ “Ph”, “C(F)(F)F” $\rightarrow$ “$\hbox {CF}_3$”) and R-group placeholders mapping attachment points to R-labels (e.g., “1*”, “2*”, ..., “10*” $\rightarrow$ “R1”, “R2”, ..., “R10”). During generation, we randomly insert abbreviation nodes or replace substructures with their abbreviated forms. We also modify bond representations by replacing single bonds with wavy or aromatic bonds and converting Kekulé structures to aromatic rings. For rendering augmentation, we introduce visual diversity through randomized parameters including font family and size (spanning standard chemical fonts to hand-drawn style fonts), bond line width, and spacing. These combined augmentations generate synthetic images with substantial structural and visual diversity across different depiction conventions, as illustrated in Fig. 4.

#### Image-level augmentation

To enhance robustness to real-world image quality variations, we apply image-level transformations simulating conditions in chemical literature. These include geometric operations (rotation, scaling, downsampling), quality degradation (Gaussian blur, salt-and-pepper noise), layout variations (adaptive cropping, random padding), and chemical document artifacts such as caption injection (scheme numbers, Roman numerals), structural noise elements (arrows, incomplete shapes, connecting lines), and marginal annotations (atom symbols, R-groups). These augmentations recreate the complex visual environment where molecular structures coexist with various annotations and artifacts in published literature.

### Unsupervised domain adaptation with self-training

Our graph reconstruction model requires rich annotations (atom coordinates and bond types) readily available in synthetic data, but typically unavailable in target domains like hand-drawn images, where only SMILES strings are provided. This necessitates unsupervised domain adaptation approaches. However, standard unsupervised domain adaptation assumes shared class labels across domains, which does not apply to molecular recognition: unlike classification tasks with fixed categories, each molecular image represents a unique chemical structure with distinct atom compositions and arrangements. This makes the typical notion of shared class-level alignment inapplicable. In our graph reconstruction setting, we consider two levels of features: atom-level and bond-level. Atom-level alignment is problematic due to class imbalance (carbon dominates), multi-token spanning (functional groups), and position-dependent features (spatial coordinates vary across domains). In contrast, bond-level features are domain-invariant, as each bond embedding encodes only structural relationships (single, double, triple, aromatic), independent of spatial layout or drawing style.

**Fig. 5:**
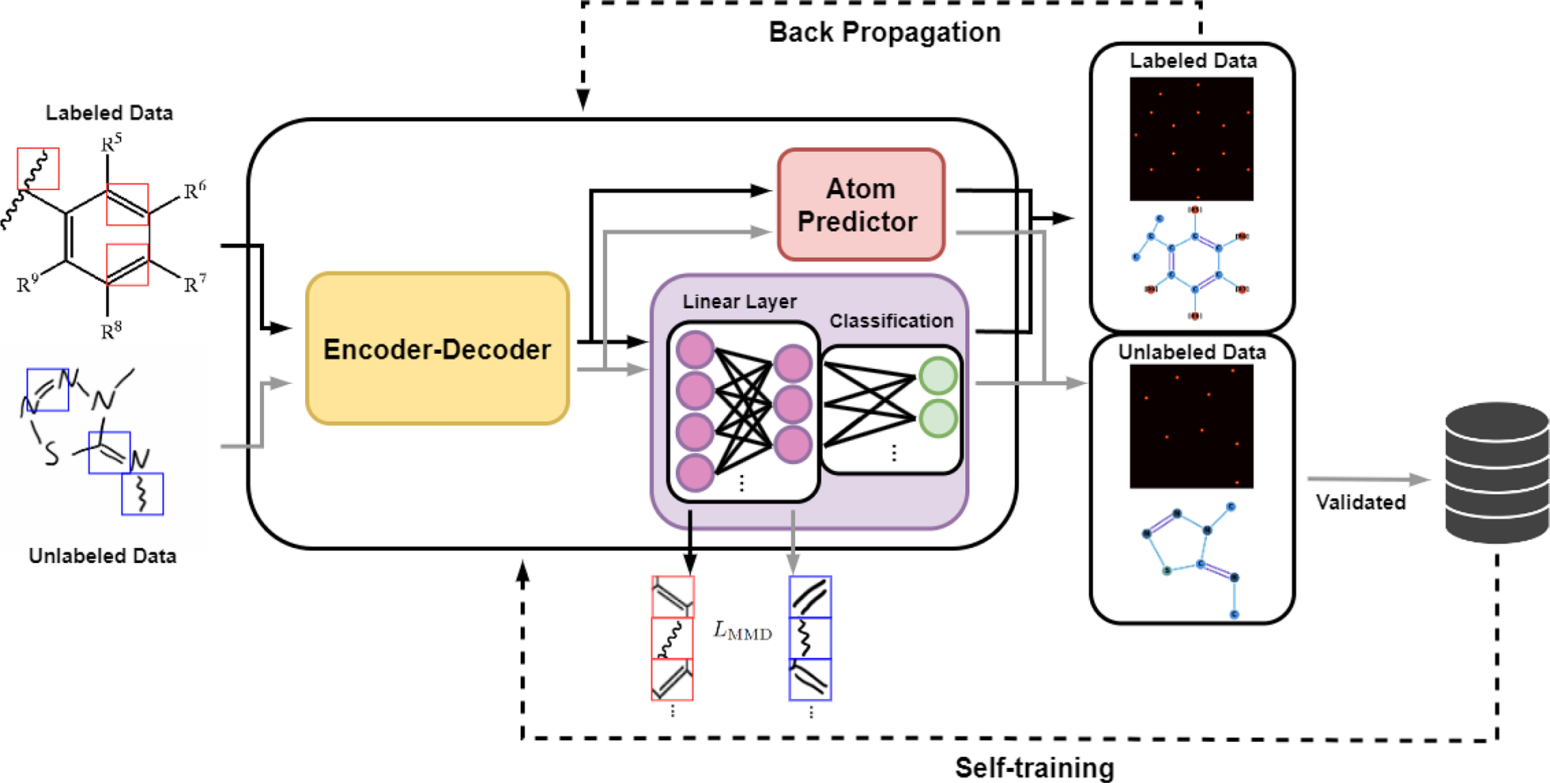
Domain adaptation pipeline. The model takes paired source (labeled) and target (unlabeled) images as input. Bond features from both domains are aligned through MMD loss. Labeled images enable supervised training via backpropagation, while validated predictions on unlabeled images generate pseudo-labels for self-training

We therefore perform unsupervised alignment at the bond level via class-conditional MMD, then apply self-training using SMILES-validated pseudo-labels to fine-tune the model on target domain data. Specifically, as shown in Fig. 5, the model processes paired images: a labeled source domain image and an unlabeled target domain image. Bond features from both images are aligned using MMD loss in the bond predictor, while the labeled image provides supervised gradients for parameter updates. Predictions on unlabeled target images are validated against ground-truth SMILES and stored as pseudo-labels for self-training. To handle visual variations (fonts, rendering styles) and structural diversity (abbreviations), we employ comprehensive data augmentation as described in Section Structure-rendering augmentation.

#### Bond-level feature alignment via MMD

We extract intermediate representations from the bond predictor before the classification layer and compute class-conditional MMD losses. We employ two strategies to ensure effective alignment. First, we apply a confidence-based filtering mechanism that retains only samples with high prediction confidence and low entropy. For source samples with ground-truth labels, we retain those correctly predicted by the model. For target samples without graph annotations, we select high-confidence predictions (low entropy). This selective approach ensures alignment focuses on reliable instances while minimizing the impact of noisy predictions. Second, we employ progressive loss weighting where MMD is weighted more heavily in early training to rapidly bridge the domain gap, then gradually reduced.

Formally, the class-conditional MMD loss is defined as

5 $$\begin{aligned} L_{\textrm{MMD}} = \frac{1}{|\mathcal {C}'|} \sum _{c \in \mathcal {C}'} MMD(F_c^{\textrm{src}}, F_c^{\textrm{tgt}}) \end{aligned}$$

where $\mathcal {C}' = \{c \mid |S_c^{\textrm{src}}| \ge \tau _1, \; |S_c^{\textrm{tgt}}| \ge \tau _1\}$ denotes the set of valid classes that satisfy the minimum sample threshold $\tau _1$. Here $F_c^{\textrm{src}}$ and $F_c^{\textrm{tgt}}$ are the high-confidence features for class *c* from the source domain and target domain, respectively. Averaging over valid classes prevents imbalance when some classes lack sufficient samples.

The MMD between two feature sets *X* and *Y* is computed with a multi-scale RBF kernel:

6 $$\begin{aligned} \begin{aligned} MMD(X,Y) = \frac{1}{|\Lambda |} \sum _{\lambda \in \Lambda } \Bigg [&\frac{1}{|X|(|X|-1)} \sum _{i \ne j} k_\lambda (x_i, x_j) \\&+ \frac{1}{|Y|(|Y|-1)} \sum _{i \ne j} k_\lambda (y_i, y_j) \\&- \frac{2}{|X||Y|} \sum _{i,j} k_\lambda (x_i, y_j) \Bigg ] \end{aligned} \end{aligned}$$

where $x_i \in X$ and $y_j \in Y$ denote individual feature vectors from the source and target domains respectively, $k_\lambda (x,y) = \exp \!\big (-\Vert x-y\Vert _2^2 / (2\lambda ^2)\big )$, and $\Lambda$ denoting the set of bandwidth parameters. This measures distribution distance via kernel embeddings: the first two terms represent within-distribution similarity, the third measures cross-distribution dissimilarity. Multiple bandwidths capture both local and global patterns.

#### Self-training with validated pseudo-labels

Following MMD alignment, we apply self-training to improve performance further. The adapted model generates predictions on unlabeled target images, producing molecular graphs with atom coordinates and bond types. These graphs are converted to SMILES strings and validated against ground-truth SMILES annotations. Only predictions that exactly match the ground-truth are retained as pseudo-labels, which we refer to as SMILES-validated predictions. These validated predictions provide complete graph supervision, which was previously unavailable in the target domain. We combine this pseudo-labeled data with the original synthetic training data and fine-tune the model for several epochs, enabling it to better capture target domain characteristics while preserving source domain knowledge.

### Postprocessing

After obtaining the predicted molecular graph, we apply rule-based postprocessing to convert it into standard molecular representations. We construct atom nodes from the predicted labels and coordinates, then connect them with bonds according to the predicted adjacency matrix. For detected abbreviations (functional groups or R-group symbols), we expand them to full substructures using the abbreviation-SMILES mappings defined in Section Structure-rendering augmentation. The resulting molecular graph is then deterministically converted to SMILES or Molfile format.

## Experiments

### Datasets

#### Training datasets

We use synthetic data for initial training. Hand-drawn and USPTO dataset for domain adaptation (MMD alignment and self-training).

***Synthetic data.*** We generate a synthetic training dataset from one million SMILES strings collected from PubChem [33] by prior work [23]. Each SMILES is converted into a molecular image with full graph annotations (atom coordinates, labels, and bond adjacency matrix) through our data generation pipeline (Section Dataset and augmentation strategies). This provides large-scale, clean supervision for base model training.

***Hand-drawn data. *** For domain adaptation, we use a publicly available hand-drawn molecular dataset [34] containing 4,070 training samples, each with a molecular image and SMILES string. We refer to this as the DECIMER training set. These samples were drawn by multiple contributors, providing diversity in drawing styles. We use this dataset for both MMD alignment and self-training to adapt the model to hand-drawn depictions. Note that this dataset contains relatively simple structures without complex abbreviations, representing a subset of real-world hand-drawn diversity.

***USPTO data.*** Additionally, we use approximately 680,000 molecular instances from the United States Patent and Trademark Office (USPTO) [35] obtained by prior work [23], which includes molecular images paired with SMILES annotations. Due to considerable noise and label inconsistencies in the automatically extracted data, we treat this as unlabeled data for self-training rather than direct supervision.

#### Evaluation datasets

We evaluate on synthetic benchmarks, literature datasets, and specialized test sets.

***Synthetic benchmarks.*** We use two synthetic test sets from prior work [23], both generated from the same 5,719 SMILES strings collected from USPTO but rendered with different toolkits. Synthetic Indigo uses the Indigo rendering engine, while Synthetic ChemDraw is drawn by the software ChemDraw.

***Literature benchmarks.*** We evaluate on six literature benchmarks containing molecular figures from scientific publications. CLEF-IP 2012 Structure Recognition Test Set (CLEF) with 992 samples was published in 2012 [36]. Maybridge UoB with 5,740 samples was created by Sadawi et al. [37]. USPTO with 5,719 samples and Japanese Patent Office (JPO) with 450 samples were obtained from the OSRA online presence [38]. STAKER with 50,000 samples consists of USPTO images downsampled to lower resolutions and was curated by Staker et al.[12]. ACS with 331 samples was collected by  Qian et al. [23].

These benchmarks span diverse image qualities, molecular complexities, and drawing styles, with JPO and ACS being particularly challenging. JPO images contain Japanese characters, exhibit non-standard drawing conventions, and suffer from poor image quality with substantial noise. ACS features greater diversity in drawing styles and extensive use of functional group abbreviations.

*Perturbed datasets.* To assess robustness to image transformations, we also evaluate on perturbed versions of four benchmarks created by Clevert et al. [39], where images are randomly rotated or sheared. CLEF_P contains 3,555 samples, JPO_P contains 1,825 samples, UOB_P contains 28,580 samples, and USPTO_P contains 24,260 samples.

*Atom localization.* We evaluate spatial localization accuracy on 1,000 molecular images with atom bounding box annotations from Oldenhof et al. [40].

*Hand-drawn test set.* We evaluate on hand-drawn molecular images from two sources: 1,018 scanned images from Brinkhaus et al. [34] with diverse drawing styles (referred to as DECIMER test set), and 613 photographed images from ChemPix [41] with varied background colors, together representing challenges in both drawing irregularity and acquisition diversity

### Evaluation metrics

#### SMILES matching

We evaluate prediction accuracy using exact SMILES match and Tanimoto similarity [42]. For exact matching, we compare the canonical SMILES of predictions and ground truth. When datasets provide both SMILES and Molfile annotations, we convert both to canonical SMILES and accept matches against either representation.

The Tanimoto similarity coefficient, computed on molecular fingerprints, measures structural similarity and assigns partial credit to near-correct predictions. Following previous work, Tanimoto similarity is calculated based on RDKit path-based topological fingerprints using the RDKit toolkit, and in line with standard practice in OCSR evaluation, we consider only the “@” chirality symbol while ignoring “/” and “$\backslash$” symbols for geometric isomerism, as most evaluation datasets lack geometric stereochemical information.

#### Atom localization

For atom localization evaluation, we predict bounding boxes for detected atoms and evaluate using precision-recall metrics at multiple IoU thresholds ranging from 0.05 to 0.35 with 0.05 increments. Since our model predicts exact atom coordinates rather than bounding boxes, we convert the coordinates to boxes by centering a fixed-size bounding box at each predicted coordinate, where the box size is computed as the average bounding box dimensions from the training subset of the localization dataset (9$\times$9 pixels). We report average precision (AP) computed with both the PASCAL VOC 11-point method [43] and the COCO all-point method [44], and summarize overall performance using mean average precision (mAP) across thresholds.

### Baseline methods

We compare against three categories of OCSR methods. *Rule-based approaches* include MolVec [10] and OSRA [11]. *Image-captioning methods* include SwinOCSR [16], Img2Mol [39], and DECIMER v2.2 [28]. *Graph reconstruction methods* include MolScribe [23], AtomLenz [29], ChemGrapher [21], and MolGrapher [20].

For more systematic comparison, we re-evaluate five recent deep learning methods using their officially released pretrained models on our evaluation datasets with unified metrics: DECIMER v2.2, MolScribe, AtomLenz, ChemGrapher, and MolGrapher. ChemGrapher is evaluated only on atom localization, as it does not produce complete molecular structures. For other methods, including MolVec, OSRA, SwinOCSR, and Img2Mol, we report results from a previous publication [23].

We focus our primary comparison with MolScribe, as it shares our graph reconstruction formulation, enabling direct methodological analysis. MolScribe uses Indigo for rendering training data, while we use RDKit. For training target representation, MolScribe tokenizes SMILES strings as sequence labels, while we extract atoms from Molfile and order them by spatial coordinates, providing more consistent ordering for complex structures. While both methods employ data augmentation, our strategy introduces more comprehensive structural and visual variations. Additionally, MolScribe directly trains on USPTO with noisy annotations, whereas we perform self-training using SMILES-validated pseudo-labels. These design choices lead to different performance characteristics across benchmarks, which we analyze in our results.

### Implementation details

We use Swin Transformer base [45] as the encoder with 384$\times$384 input resolution. The decoder is a 6-layer Transformer [46] with 8 attention heads, hidden dimension 256, sinusoidal positional encoding, and dropout 0.1. The projection layer and atom predictor are single feed-forward layers. The bond predictor uses an 8-head multi-head attention mechanism to attend from decoder outputs to projection layer outputs, producing enriched atom features. These are combined with the original decoder outputs through a learnable weighted residual connection, then passed through a two-layer feed-forward network to predict bond types.

The base model is trained on 1 M synthetic samples for 30 epochs using Adam optimizer with batch size 64, maximum learning rate $4 \times 10^{-4}$, 5% linear warmup, and cosine decay. The heatmap loss weight is set to 0.5 and label smoothing to 0.05.

Domain adaptation proceeds in three steps. First, we self-train on USPTO for three iterations of 3 epochs each, yielding 259K pseudo-labels. Second, we apply MMD alignment on hand-drawn data for 5 epochs with batch size 32 and learning rate $5 \times 10^{-5}$. The MMD weight follows a progressive schedule: 0.1 in epoch 0, 0.075 in epoch 1, and 0.05 thereafter. We filter bond features to retain predictions with confidence above 0.95 and entropy below 0.1, increasing to 0.98 and 0.05, respectively, after the first epoch. MMD kernel bandwidths are set to $\{0.25, 0.5, 1.0\}$ for non-bond features, $\{0.5, 1.0, 1.5\}$ for chirality bonds, and $\{0.3, 0.6, 1.2\}$ for other bond types. Third, we self-train on hand-drawn data for five iterations, yielding 3,671 pseudo-labels. All fine-tuning stages use batch size 64 and learning rate $5 \times 10^{-5}$ with the same warmup and decay schedule. Training is conducted on two NVIDIA A100 GPUs with 40GB of memory each.

## Results and discussion

### Quantitative results

In this section, we present the evaluation of our model on the benchmark datasets and provide a comparative analysis against other baseline methods.

#### Robust hand-drawn recognition via domain adaptation

**Table 1:** Performance comparison on the target domain (DECIMER test set) and out-of-domain ChemPix benchmark. Results show both exact match accuracy in % (where Tanimoto similarity equals 1) and average Tanimoto similarity ($\overline{T}$)

	DECIMER test set		ChemPix	
Method	Acc.($T=1$)	$\overline{T}$	Acc.($T=1$)	$\overline{T}$
Image captioning				
DECIMER v2.2	*71.9*	*85.0*	*51.4*	** 73.6**
Graph reconstruction				
MolScribe	10.1	30.4	26.1	49.0
AtomLenz	30.0	53.5	48.4	69.4
MolGrapher	10.7	26.3	14.5	17.0
**AdaptMol (ours)**	**82**.**6**	**88**.**5**	**60**.**5**	*73.4*

Bold represents the best performance and italics represents the second-best performance

Table 1 shows results on hand-drawn molecular images. On the DECIMER hand-drawn test set, our method achieves 82.6% accuracy, outperforming DECIMER-Handdraw (71.9%) by 10.7 percentage points. Compared to AtomLenz (32.1%), the improvement is particularly significant, with an absolute improvement of 50.5%, despite both methods employing self-training on the same training data. This substantial gap validates our hypothesis that base model accuracy is critical for self-training success. Our MMD alignment and data augmentation first strengthen the base model to generate high-quality pseudo-labels, whereas AtomLenz’s exhaustive correction approach cannot compensate for insufficient base model accuracy. Notably, we achieve these results using only 4,080 authentic hand-drawn images for domain adaptation, whereas DECIMER-Handdraw requires generating over 38 million synthetic hand-drawn images, demonstrating substantially better data efficiency when training on real-world data.

On ChemPix, which contains photographed images with diverse backgrounds, our model achieves approximately 10 percentage points improvement over the previous best method without additional adaptation on this dataset. This demonstrates that our adaptation framework generalizes across different hand-drawn sources and acquisition conditions. The strong cross-dataset performance indicates that the representations learned through our domain adaptation pipeline capture fundamental drawing irregularities rather than overfitting to the specific characteristics of the DECIMER training set.

#### Strong generalization across diverse datasets

**Table 2:** Performance comparison across realistic and synthetic datasets

Methods	Realistic						Synthetic	
	CLEF	JPO	UoB	USPTO	Staker	ACS	Indigo	ChemDraw
Rule-based								
$\hbox {OSRA}^{p}$	84.6(88.9)	55.3(70.5)	78.5(90.4)	87.4(94.1)	0.0(17.4)	55.3(63.0)	95.0(98.1)	87.3(94.8)
$\hbox {MolVec}^{p}$	82.8(88.5)	67.8(85.0)	80.6(91.5)	88.4(95.8)	0.8(23.8)	47.4(66.7)	95.4(98.6)	87.9(95.9)
Image captioning								
$\hbox {Img2Mol}^{p}$	18.3(74.1)	16.4(48.5)	68.7(84.7)	26.3(72.1)	17.0(63.0)	23.0(49.6)	58.9(91.0)	46.4(84.3)
$\hbox {SwinOCSR}^{p}$	30.0(59.4)	13.8(49.3)	44.9(90.4)	27.9(65.8)	-(–)	27.5(41.7)	74.0(89.3)	79.6(90.9)
DECIMER v2.2	77.7(91.1)	75.7(91.0)	87.2(96.2)	59.6(92.9)	66.3(93.1)	37.7(65.4)	**97**.**8**(99.6)	**97**.**8**(99.4)
Graph reconstruction								
MolScribe	*87.5*(92.3)	*78.8*(91.0)	*88.2*(95.6)	**92**.**6**(98.0)	**84**.**4**(97.8)	*72.8*(77.3)	*97.7*(98.9)	93.8(96.9)
AtomLenz	2.7(51.1)	26.73(49.8)	40.8(66.6)	2.4(40.0)	0.0(0.6)	6.0(24.2)	7.1(68.8)	4.5(59.9)
MolGrapher	57.2(88.7)	73.0(84.4)	85.1(93.6)	74.9(94.5)	0.0(8.1)	41.0(62.8)	79.6(97.0)	79.8(96.1)
**AdaptMol**	**92**.**7**(92.6)	**88**.**2**(94.1)	**89**.**3**(95.8)	*90.9*(97.2)	*84.0*(96.6)	**75**.**5**(79.5)	95.6(98.1)	*95.9*(98.3)

Main scores are exact matching accuracy in % (Tanimoto similarity = 1). Values in parentheses denote average Tanimoto similarity in %. - means not available. ^*p*^: results from [23]

Table 2 shows results on molecular figures from scientific literature and synthetic datasets. Our method achieves the best performance on four out of six literature benchmarks: CLEF (92.7%), JPO (88.2%), UOB (89.3%), and ACS (75.5%), outperforming the second-ranked MolScribe with absolute improvements of 5.2%, 9.4%, 1.1%, and 2.7%, respectively. These improvements stem from two key design choices. Our coordinate-based target representation orders atoms by spatial positions, whereas MolScribe’s SMILES tokenization approach follows the atom ordering in SMILES strings. This provides more consistent and predictable sequence ordering and reduces ambiguity during the model’s learning, particularly for complex molecular structures with multiple branches or rings. Additionally, our self-training with SMILES-validated pseudo-labels filters noise such as label inconsistencies from the USPTO training set, yielding cleaner training signals that improve performance across benchmarks, and we will further discuss this in ablation studies. However, on USPTO and Staker benchmarks, MolScribe achieves slightly higher accuracy by directly training on all 680K USPTO samples with automatically extracted annotations. Despite the noise in these annotations, MolScribe may benefit from overfitting to the specific characteristics of USPTO data, as both training and test sets originate from the same source. This advantage extends to Staker, which consists of USPTO images at reduced resolutions, sharing the same underlying data distribution.

For synthetic benchmarks, DECIMER achieves top performance on both Indigo and ChemDraw by training on 400 million samples rendered with multiple tools, including Indigo and ChemDraw, making both test sets in-distribution. Our model trains exclusively on 1 million RDKit-rendered images, making both benchmarks out-of-distribution. We achieve second place on ChemDraw Synthetic and third on Indigo Synthetic. MolScribe, which uses Indigo rendering for training, outperforms us on Indigo Synthetic by benefiting from rendering tool alignment. However, on ChemDraw Synthetic, where both our model and MolScribe face out-of-distribution conditions, we outperform MolScribe. This demonstrates the effectiveness of our comprehensive data augmentation strategy in enabling cross-rendering generalization even without exposure to the target rendering style during training.

Taken together with the results presented earlier, our model achieves the best reported performance on hand-drawn datasets while remaining competitive across literature and synthetic benchmarks, demonstrating consistent generalization across diverse visual styles.

#### Robustness to geometric transformations

**Table 3:** We report the percentage of perfectly recognized molecule images on perturbed datasets from real scientific documents

Method	USPTO_P	UoB_P	CLEF_P	JPO_P
DECIMER v2.2	66.6	**88**.**5**	*84.4*	69.8
MolScribe	**92**.**6**	86.6	93.7	*69.9*
AtomLenz	10.9	49.7	16.0	24.6
MolGrapher	72.7	84.0	59.3	57.8
**AdaptMol (Ours)**	*91.8*	*87.9*	**94**.**2**	**82**.**5**

Bold represents the best performance and italic represents the second-best performance

To further demonstrate the robustness of our model, we evaluate on perturbed datasets created by Clevert et al. [39], where images undergo random rotation and shearing transformations. As shown in Table 3, our method ranks first on two out of four benchmarks: CLEF_P (94.2%) and JPO_P (82.5%). On JPO_P, we outperform the second-best method by over 12%, demonstrating particularly strong robustness to geometric transformations. DECIMER achieves the best performance on UOB_P. This can be attributed to the original UOB dataset’s relatively uniform distribution and lack of certain noise types present in other benchmarks, making it more aligned with synthetic molecular distributions and closer to DECIMER’s massive training data distribution. On USPTO_P, MolScribe maintains its advantage, consistent with our previous analysis, as it directly trains on large-scale USPTO data. Although we do not rank first on these two benchmarks, our model achieves a competitive second-place performance with small margins, demonstrating strong robustness across diverse perturbation scenarios.

#### Accurate atom localization

Since our model is a graph reconstruction-based approach, accurately localizing atoms is fundamental to generating correct molecular graphs. We evaluate atom localization performance to assess the spatial prediction accuracy of different graph reconstruction models. Table 4 shows results on the dataset from Oldenhof et al. [40]. Our method achieves the best performance under COCO-style evaluation with 99.00% mAP, which computes the area under the full precision-recall curve rather than sampling at fixed recall levels, providing a more comprehensive assessment of localization quality compared to the traditional 11-point interpolation method. We also achieve the highest average recall (99.53%) across IoU thresholds, with average precision (99.43%) only 0.01 percentage points lower than the best method. These results demonstrate strong atom localization capability, with near-perfect precision and recall, indicating that false positives and false negatives rarely occur in our prediction.

**Table 4:** Atom localization performance comparison. mAP is computed using IoU thresholds of [0.05, 0.1, 0.15, 0.2, 0.25, 0.3, 0.35], reported in both COCO-style and 11-point interpolated formats

	mAP	mAP	Average	Average
Methods	(COCO)	(11pt)	Prec	Recall
AtomLenz	79.85	80.59	97.92	83.17
MolGrapher	94.47	87.95	96.20	97.57
ChemGrapher	91.68	**90**.**47**	**99**.**44**	92.56
MolScribe	*97.54*	89.77	98.09	*98.92*
**AdaptMol**	**99**.**00**	*90.46*	*99.43*	**99**.**53**

Precision measures the percentage of correctly detected atoms among all predictions, while recall measures the percentage of ground truth atoms that are successfully detected

### Qualitative results

#### Visualization of prediction

**Fig. 6:**
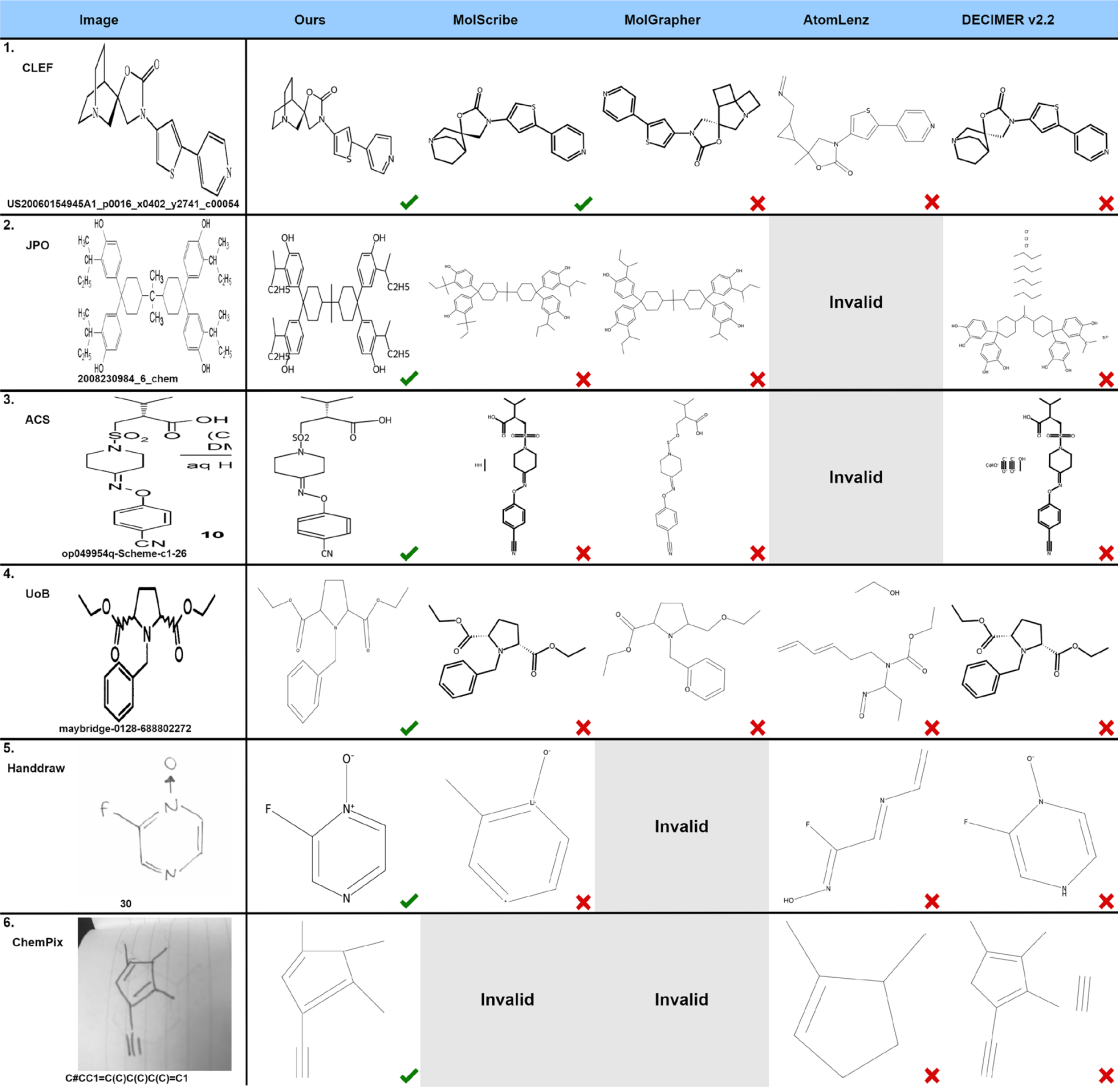
Visualization of predictions on diverse benchmarks compared to other SOTA methods. Our method (column 2) displays reconstructed graphs with preserved spatial layout; other methods are generated from predicted SMILES using RDKit. Original image IDs from the test datasets are provided beneath each input image to facilitate cross-referencing

We conduct a qualitative evaluation comparing our method against other approaches. Figure 6 presents representative cases from diverse benchmarks, with our predictions shown in the second column.

Row 1 shows a CLEF example demonstrating our model’s capability to handle complex overlapping molecular structures. Row 2 presents a JPO case featuring explicit hydrogen atoms, a common notation in patent documents. Unlike typical implicit hydrogen representations, recognizing when to retain explicit hydrogens requires chemical knowledge, which our model successfully captures. Row 3 displays an ACS example containing additional captions and incomplete structures, showcasing our model’s robustness to image-level noise and text interference. Row 4 highlights a rare case where a wavy bond is drawn with sharp angular segments rather than smooth waves. Despite the absence of such cases in our training data, our model correctly predicts it, demonstrating strong inter-class discrimination learned through MMD alignment.

Row 5–6 feature hand-drawn molecules from the DECIMER test set and ChemPix. The DECIMER example showcases not only accurate recognition of hand-drawn structures but also proper handling of arrow symbols, while the ChemPix case, an out-of-distribution dataset, demonstrates the excellent generalization capability of our model. Notably, while graph reconstruction methods including ours can preserve spatial layouts of atoms and bonds, visualizing these reconstructed graphs requires complex rendering procedures. For simplicity, we render predictions from other methods (MolScribe, MolGrapher, AtomLenz) through their predicted SMILES strings using RDKit. In contrast, we directly display our reconstructed molecular graphs, showcasing the predicted atom positions and bond layouts.

#### Error case analysis

**Fig. 7:**
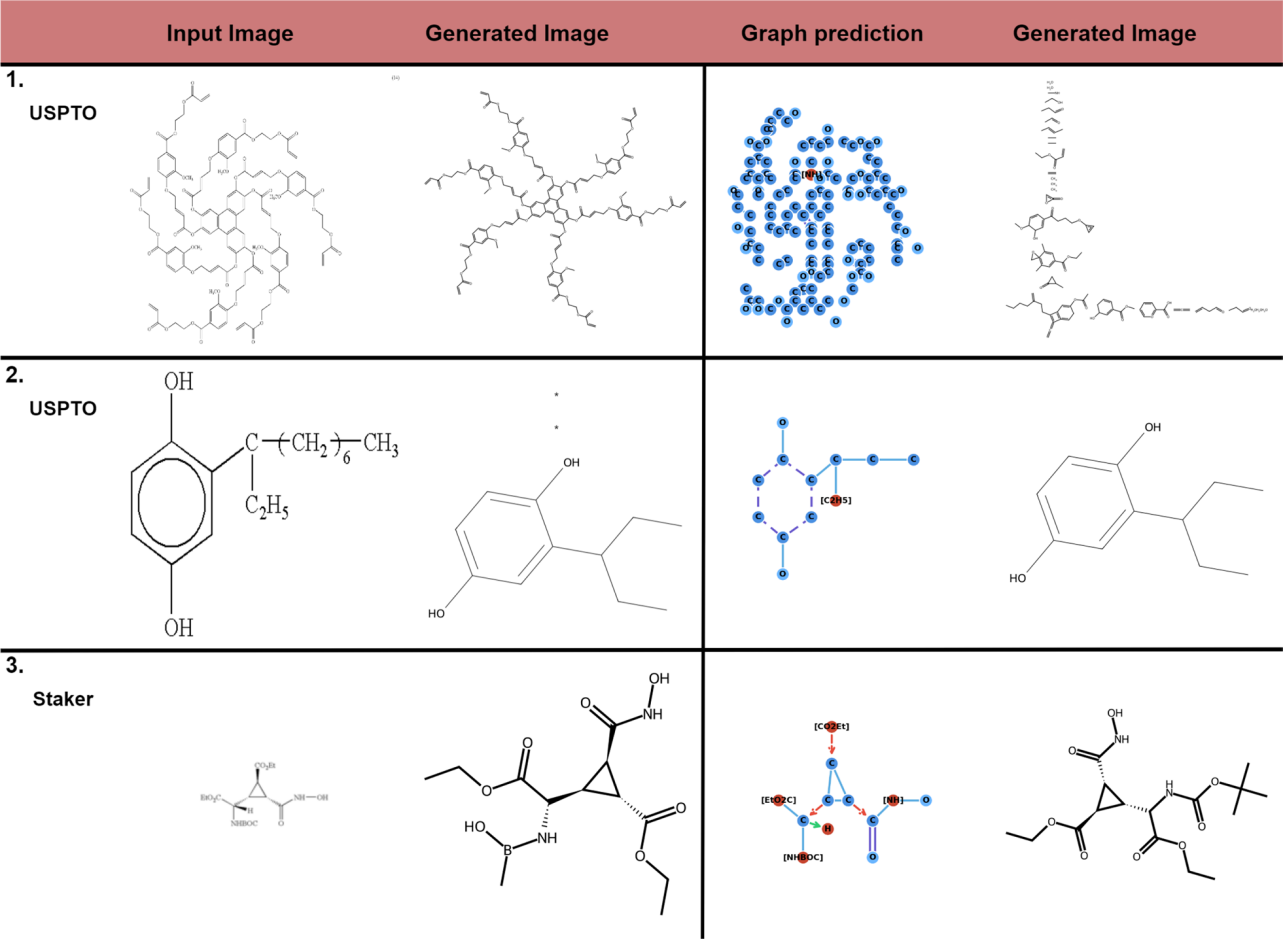
Visualization of predictions on error cases

To better understand our model’s limitations, we analyze three representative failure cases in Fig. 7. The figure shows input images (column 1), ground truth structures rendered from SMILES via RDKit (column 2), our predicted graph structures (column 3), and our predicted SMILES rendered via RDKit (column 4).

First, our model struggles with extremely large molecular structures. Row 1 shows a molecule with over 120 atoms whose original resolution far exceeds our model’s input size. After resizing, the image becomes blurred with significantly fewer pixels per atom or bond compared to simpler molecules, leading to recognition errors.

Second, our model fails to accurately predict Markush structures. In Row 2, the notation ($\hbox {CH}_2\hbox {)}_6$ should be recognized with each parenthesis as a separate atom, labeled as [(] or [)] in the SMILES representation. However, our synthetic training data lacks such cases, preventing the model from learning this notation.

Third, stereochemistry information is lost when stereo bonds connect to abbreviated functional groups. Row 3 demonstrates this issue: while the graph structure is correctly recognized (column 3), the final output (column 4) lacks a stereo bond present in the ground truth (column 2). This occurs during RDKit post-processing, as expanded abbreviations lack the atomic coordinates necessary for chirality assignment. Directly assigning chirality to abbreviated terms also fails, as it is lost upon expansion. We hope future work can develop alternative post-processing methods to address this limitation.

### Ablation study

In this section, we conduct an ablation study to present the importance of each component in our pipeline.

#### Data augmentation

**Table 5:** Impact of data augmentation strategies on model performance across diverse benchmarks

Model	Indigo	JPO	ACS	UOB	JPO_P	CLEF_P
w/o Structure-rendering Aug	93.4	66.1	45.9	84.8	60.1	91.6
w/o image Aug	93.7	85.9	72.5	88.4	78.9	92.2
AdaptMol	95.6	88.2	75.5	89.3	82.5	94.2

We evaluate models without structure and rendering augmentation, without image augmentation, and our full model with comprehensive augmentation

As shown in Table 5, we categorize data augmentation into two types, namely structure-rendering augmentation and image-level augmentation. To analyze their contributions, we trained two comparative models, one without structure and rendering augmentation and the other without image-level augmentation. The model trained without structure-rendering augmentation exhibits a substantial performance decline on challenging datasets with complex abbreviations. On JPO, accuracy drops by approximately 20 %, and on ACS by 30 %. In contrast, on datasets without abbreviations, such as UOB and the synthetic Indigo benchmark, the decline is minimal at 3.5 and 2.2 % respectively. This discrepancy arises because RDKit, by default, cannot generate abbreviated functional groups. Without structure augmentation that introduces abbreviations during training, the model fails to learn representations for these commonly used notations in real scientific literature. For image-level augmentation, which involves transformations such as rotation, scaling, and noise injection, the model shows only slight performance decline on clean datasets. For example, accuracy drops by 2.3 % on JPO. However, on perturbed datasets, the impact is more pronounced, with a 4.4 % drop on JPO_P. This demonstrates that image-level augmentation is particularly crucial for robustness to geometric perturbations and image quality variations. Taken together, these results indicate that structure-rendering augmentation serves as the more fundamental enhancement, enabling the model to understand diverse molecular structures and notations prevalent in scientific literature. While image-level augmentation has less dramatic impact on clean datasets, it significantly improves robustness to geometric perturbations and image quality degradation. Both augmentation strategies demonstrate their importance in enhancing the model’s generalization capability and robustness across diverse real-world conditions.

#### Heatmap supervision

**Table 6:** Impact of heatmap supervision on challenging benchmarks with poor image quality and perturbations

Model	JPO	JPO_P	CLEF_P
w/o heatmap	82.1	77.7	91.4
AdaptMol	$\hbox {88.2}_{+6.1}$	$\hbox {82.5}_{+4.8}$	$\hbox {94.2}_{+2.8}$

**Table 7:** Impact of dual position representation on atom localization performance across different IoU thresholds

	0.05		0.1		0.15		0.2		0.25		0.3		0.35		mAP	Average Recall
	**Prec**	**FP**	**Prec**	**FP**	**Prec**	**FP**	**Prec**	**FP**	**Prec**	**FP**	**Prec**	**FP**	**Prec**	**FP**		
w/o heatmap	98.61	356	98.59	362	98.55	370	98.50	385	98.33	427	97.94	527	96.75	832	97.91	99.51
AdaptMol	99.87	33	99.85	38	99.82	45	99.78	55	99.64	92	99.23	194	97.88	535	99.00	99.53

**Fig. 8:**
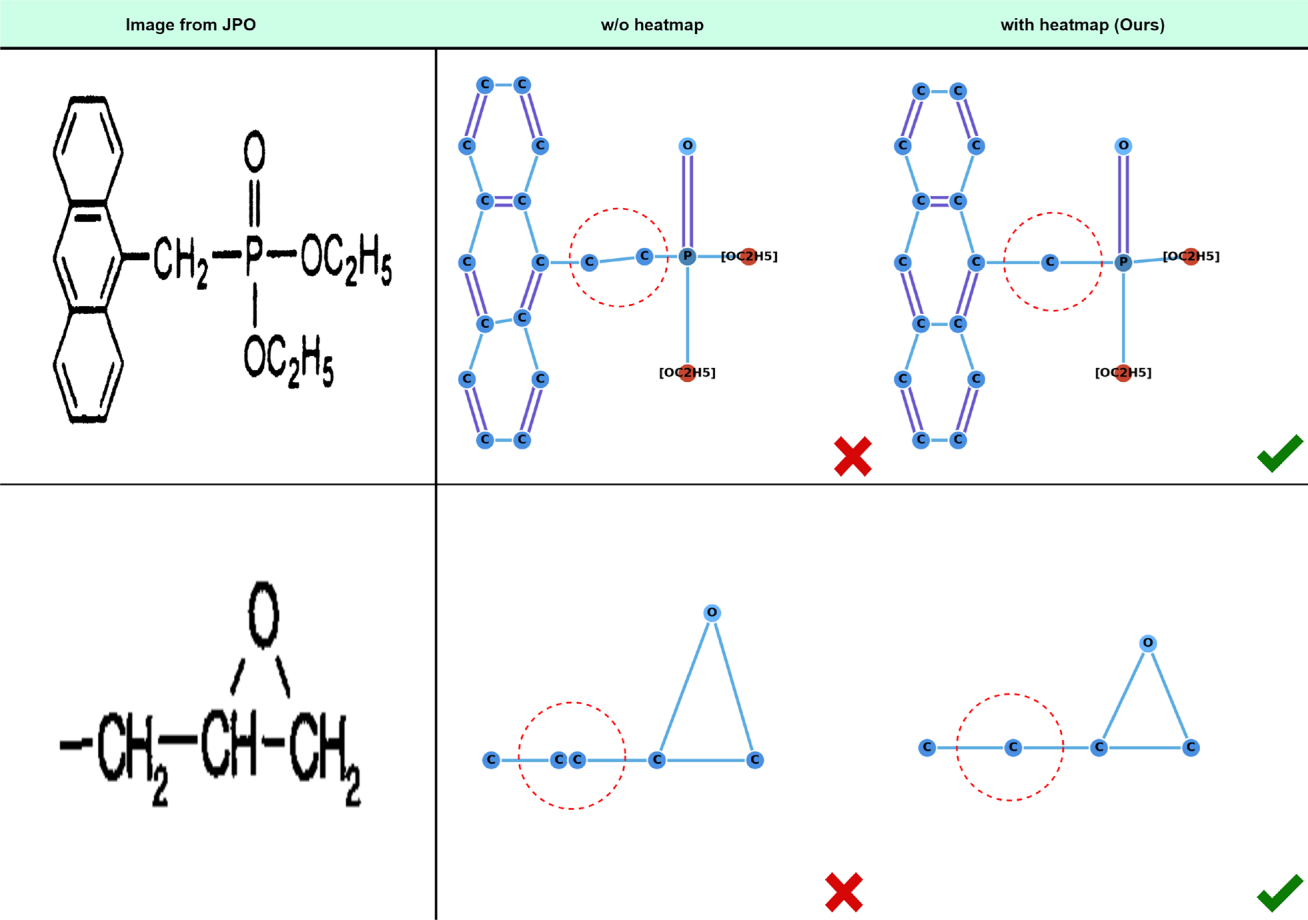
Comparison of models with and without heatmap representation. The model with heatmap (right) correctly reconstructs the structure, while the model without heatmap (center) consistently predicts an extra C atom

In previous graph reconstruction models, coordinate prediction is supervised solely through cross-entropy on discrete coordinates. In contrast, we introduce an additional heatmap-based supervision. To assess its effect, we retrained a model without heatmap supervision. Table 6 presents the evaluation results of models with and without heatmap supervision. On the JPO dataset, the model trained without heatmap supervision shows a substantial performance drop. As illustrated in Fig. 8, the absence of heatmap guidance leads to a higher probability of predicting redundant atoms (false positives). We therefore conducted an atom localization evaluation on this model. As shown in Table 7, removing heatmap supervision leads to consistently lower precision and more false positives across all IoU thresholds, while recall remains unchanged, resulting in a lower overall mAP (97.91 vs. 99.00). These results indicate that the heatmap loss not only provides additional spatial constraints during training but also offers two notable advantages. First, it supplies weakly supervised localization on top of sequence prediction, enabling the model to better align atomic distributions in the two-dimensional plane and bridging the gap between sequential prediction and spatial structure. Second, as an aggregated probabilistic representation, the heatmap smooths token-level prediction noise to some extent, thereby enhancing robustness in position prediction.

#### Overall pipeline

To validate the effectiveness of our approach, we conduct ablation studies on the components we introduced in the pipeline. Table 8 shows results for five model variants: (1) base model without augmentation, MMD, or self-training; (2) with hand-drawn font augmentation; (3) with augmentation and MMD alignment; (4) our full pipeline as described in Sect. Implementation details and (5) retrained baseline trained from scratch on synthetic data plus pseudo-labels for 30 epochs (no MMD or self-training). Results show that hand-drawn font augmentation improves accuracy by approximately 20%, demonstrating the importance of bridging the atomic symbol appearance gap between synthetic and hand-drawn images. And bond level MMD alignment further provides an additional 12% improvement.

**Table 8:** Domain adaptation pipeline ablation on hand-drawn and literature benchmarks

Model	Hand-drawn	ChemDraw	JPO	JPO_P
Base model	10.4	92.3	82.7	74.4
+ Font augmentation	$\hbox {30.2}_{+19.8}$	92.5	82.8	75.5
+ Font Aug + MMD	$\hbox {42.1}_{+11.9}$	94.0	83.0	75.7
+ Font Aug + MMD + self-training (ours)	$\hbox {82.6}_{+40.5}$	95.9	88.2	82.5
Retrained baseline	87.2	$\hbox {94.4}_{-1.5}$	$\hbox {87.0}_{-1.2}$	$\hbox {80.2}_{-2.3}$

Models are progressively built by adding components. The retrain baseline trains from scratch on synthetic data plus pseudo-labels for 30 epochs without MMD alignment

The retrain baseline achieves higher accuracy (87%) on the hand-drawn test set compared to our full pipeline (82%). This can be attributed to extended training (30 epochs), allowing the retrain model to better fit the hand-drawn distribution, whereas our pipeline uses only 5-epoch fine-tuning to preserve knowledge from synthetic pre-training. However, this reveals an important trade-off. While the retrain baseline optimizes for hand-drawn performance through extended training, it lacks the cross-domain robustness provided by MMD alignment. Performance on synthetic and literature benchmarks demonstrates this limitation. Our pipeline outperforms the retrain baseline on both types of benchmarks, indicating that MMD alignment enhances generalization not only to the hand-drawn target domain but also across diverse molecular image sources.

**Fig. 9:**
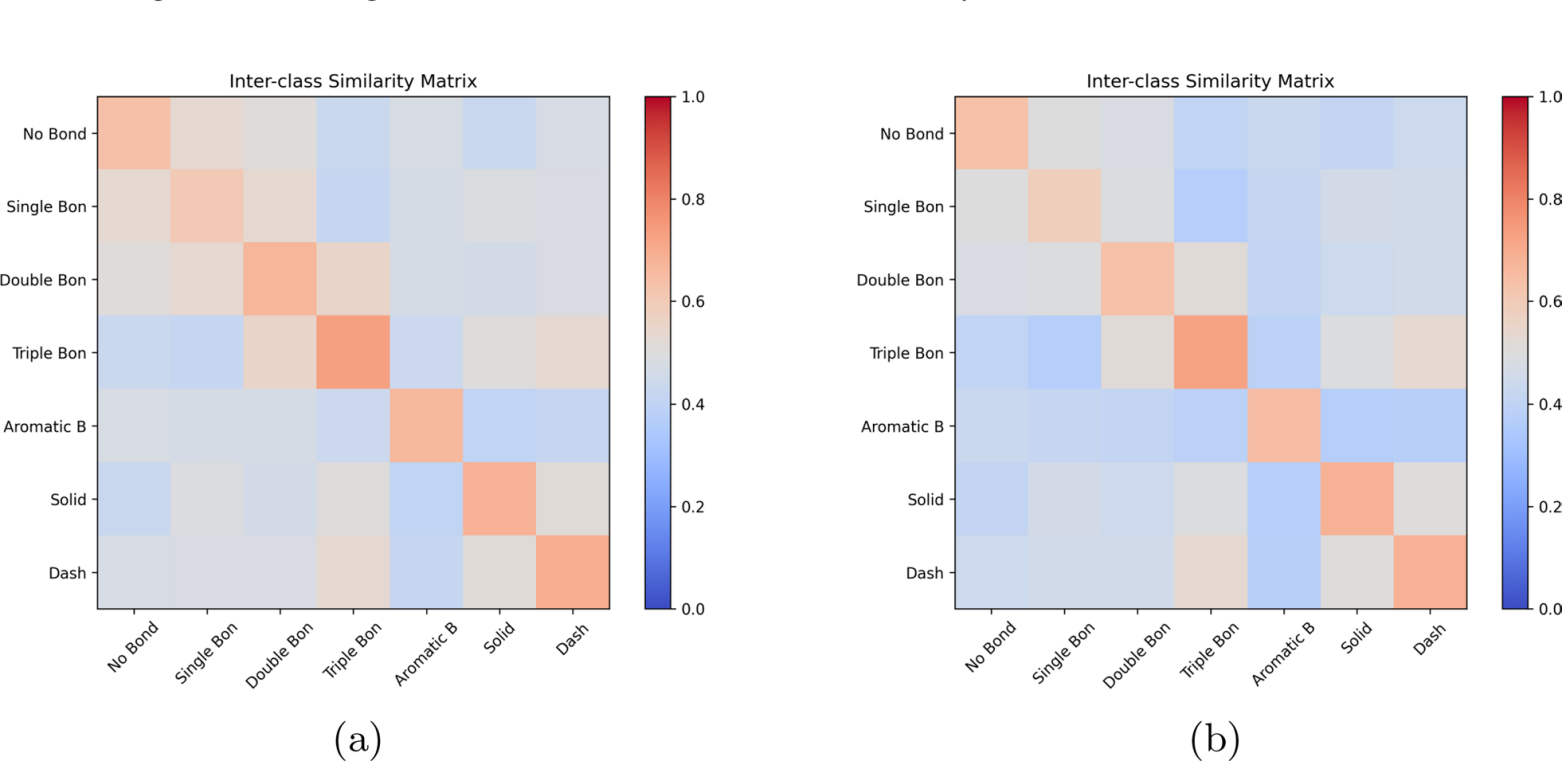
Bond class similarity matrices **a** before and **b** after MMD alignment. Blue indicates low similarity. MMD alignment substantially reduces inter-class similarity (off-diagonal regions), improving bond type discrimination

**Fig. 10:**
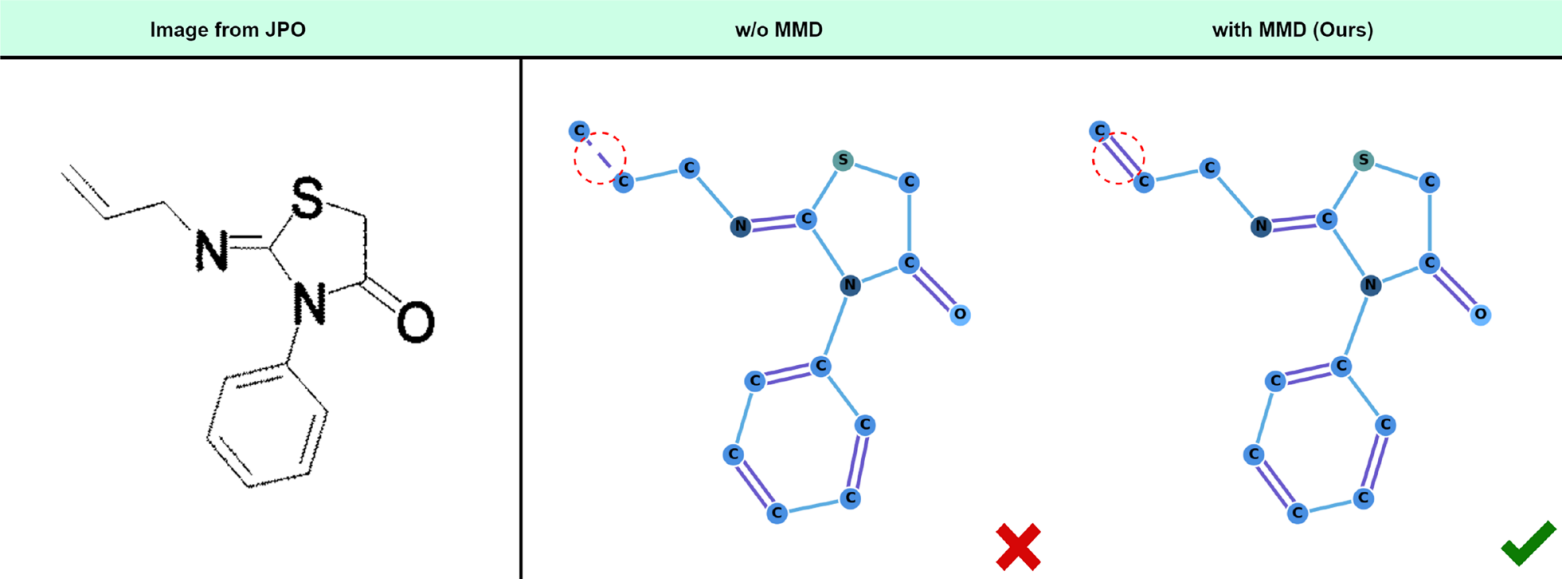
Comparison of models with and without MMD. The model with MMD (right) correctly reconstructs the structure, while the model without MMD (center) wrongly predicts the jagged double bond as an aromatic bond

We investigate the mechanism behind MMD’s generalization benefit by analyzing learned bond representations. The MMD loss, while primarily aligning bond class embeddings with target domain data, also implicitly promotes inter-class separation. Figure 9 shows similarity matrices between bond classes before and after MMD alignment. Before MMD (left), several bond classes exhibit high inter-class similarity. After MMD (right), inter-class similarity decreases substantially, with more prominent blue regions indicating better class discrimination. Figure 10 provides qualitative validation through prediction examples: without MMD alignment, the model misclassifies low-resolution jagged double bonds as aromatic bonds, whereas with MMD these errors are corrected, confirming improved discriminative ability.

Overall, these ablations demonstrate that each component contributes to the pipeline’s effectiveness: hand-drawn font augmentation addresses atomic symbol appearance differences, MMD alignment on bond features improves inter-class discrimination and cross-domain generalization, and self-training effectively leverages unlabeled target data for complete molecular graph supervision, together enabling robust cross-domain recognition.

#### Direct training versus self-training on USPTO

**Table 9:** Comparison between direct training on USPTO with automatically extracted annotations versus our SMILES-validated self-training approach

Training strategy	CLEF	JPO	ACS	USPTO	Staker
Direct training (680K samples)	89.5	82.1	66.7	**92**.**1**	**84**.**4**
Self-training (259K, Ours)	**92**.**7**	**88**.**2**	**75**.**5**	90.9	84.0

Direct training uses all 680K samples, while self-training uses 259K validated pseudo-labels

To further validate our self-training strategy, we compare two approaches for leveraging USPTO data: (1) direct training on the full 680K USPTO samples with automatically extracted graph annotations, and (2) our self-training approach using 259K SMILES-validated pseudo-labels. Table 9 shows performance across benchmarks. As expected, direct training achieves higher accuracy on USPTO (92.1%) and Staker (84.4%) compared to our self-training approach (90.9% and 84.0%, respectively). This advantage likely stems from overfitting to the specific characteristics of USPTO data, as both training and test sets originate from the same source.

However, on other benchmarks including CLEF, JPO, and ACS, our self-training approach consistently outperforms direct training. We attribute this to label inconsistencies in the USPTO training set caused by inconsistent functional group representations. Our synthetic training data uses abbreviated forms for functional groups (e.g., COOH for carboxyl, Ph for phenyl), while USPTO automatically parsed annotations contain both abbreviated and expanded representations. For example, the label for functional group node $\hbox {CF}_3$ is always “CF3” in synthetic data, but USPTO may produce “CF3”, “CFFF”, or “FCFF” depending on the source. This inconsistency causes the direct training model to encounter conflicting supervision: the same visual pattern corresponds to different atom sequences across training samples. During inference, this confusion leads the model to generate duplicated atoms or completely incorrect structures. Our SMILES-validated self-training filters such inconsistencies, retaining only predictions that produce valid molecular structures.

**Fig. 11:**
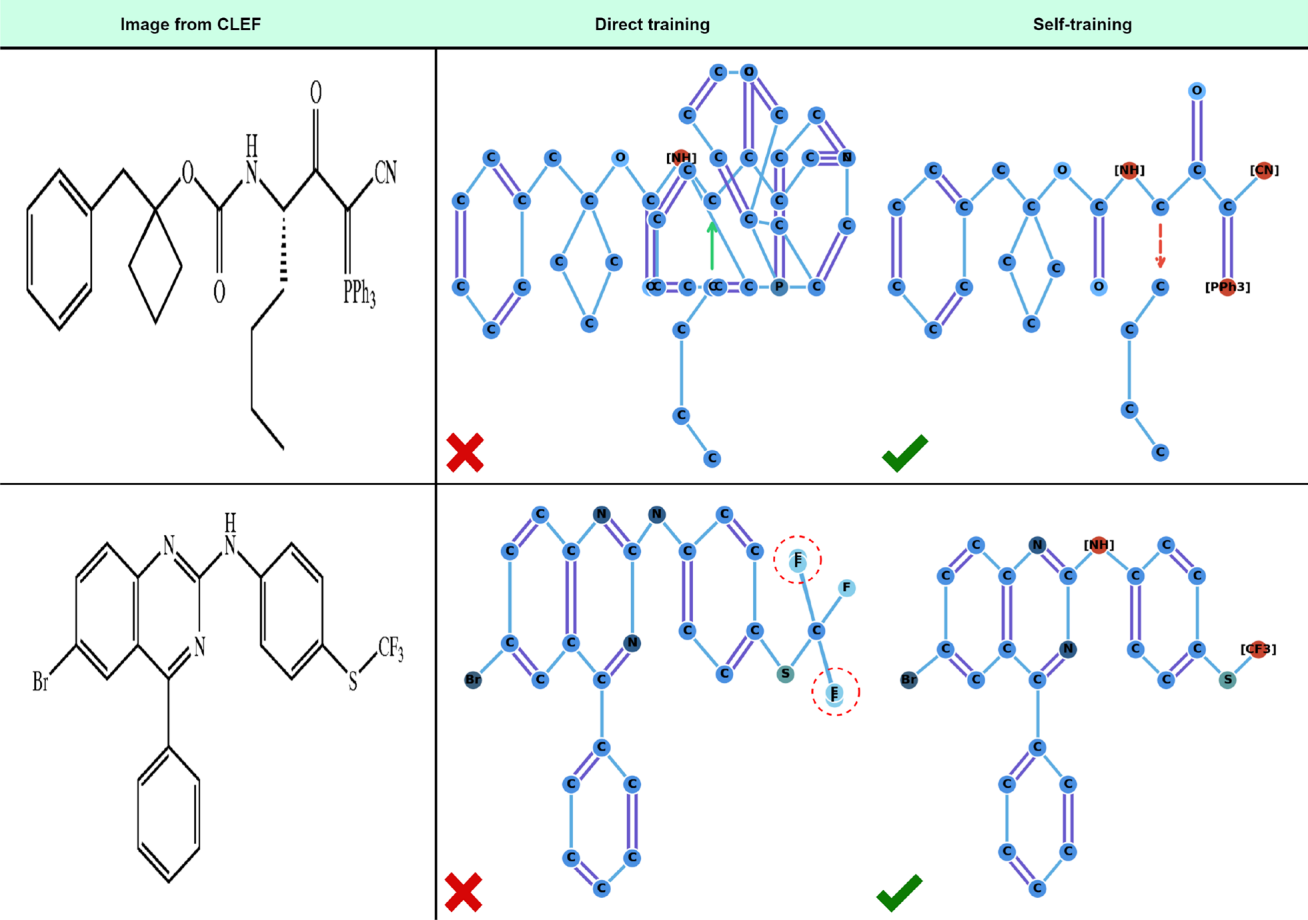
Comparison of model predictions on image with abbreviations. Self-training (right) correctly reconstructs the structure while direct training (center) produces duplicated atoms or wrong structures. Abbreviation predicted by the model will be correctly transferred to the chemical structure by post-processing

Figure 11 presents qualitative examples comparing predictions from both approaches on molecules with functional group abbreviations. The direct training model produces duplicated or incorrect structures due to conflicting training signals, while our self-training approach generates correct predictions by learning from consistent, validated supervision. These results demonstrate that data quality, not just quantity, is critical for effective molecular graph recognition.

### Future work

Our model performs strongly on the hand-drawn dataset and remains competitive on molecular figures from the literature. Nevertheless, several limitations remain concerning both the model architecture and its ability to handle diverse molecular structures. From the architectural perspective, we adopt a sequence generation strategy to predict all atoms and their positions in molecular graphs. This approach is efficient and enables end-to-end multitask bond prediction, yet it is constrained by the limited sequence length, which prevents the model from accurately predicting molecules containing a large number of atoms. This limitation might be mitigated by first localizing atoms and then splitting the sequence into smaller segments, a direction that warrants further investigation in future work. In addition, our model shows a poor ability to recognize some of the Markush structures, particularly those involving notations such as “[]” and “()” used to represent repeating units, as reflected in some cases within the USPTO dataset. This issue arises because our synthetic data generation pipeline cannot produce such structures. Although we employed prediction-based finetuning on the USPTO training set, the base model itself failed to correctly predict these cases, and finetuning consequently did not provide any improvement. Addressing this limitation may require alternative data generation strategies that better capture such specialized structures.

### Implementation notes

The maximum output sequence length in our model is set to 480 tokens. Under our tokenization scheme, each atom requires at least 3 tokens, which implies a theoretical upper bound of about 160 atoms when all atoms are represented by single character symbols such as C. In practice, this upper bound is lower because many atoms or groups are represented using multi character abbreviations, which consume more tokens. In addition, all input images are resized to 384 $\times$ 384 before inference. For molecules with many atoms, this resizing step may increase spatial crowding and reduce the visual separability of structural elements, which can in turn affect recognition accuracy. Therefore, although the token budget provides a rough estimate of the maximum predictable SMILES length, the effective limit also depends on molecular notation and image complexity. A more systematic analysis of this limitation is left for future work.

## Conclusion

In this study, we introduced a new model AdaptMol  for the task of optical chemical structure recognition (OCSR). The model is first trained on purely synthetic datasets and then enhanced through our simple yet efficient pipeline, which combines MMD-based domain adaptation with pseudo-label finetuning. This approach improves performance on hand-drawn molecular images substantially without sacrificing accuracy on molecular figures from the literature. By successfully adapting to the hand-drawn domain, our pipeline shows promise for extending to other domains at low cost, thereby enabling more accurate automated parsing of molecular figures from diverse literature sources, which is an essential component in building comprehensive chemical information extraction systems. Extensive experiments confirm the strong generalization ability and robustness of our model under various noise conditions. Nevertheless, certain limitations remain, which will be the focus of future improvements.

## Data Availability

The source code of this article is available at https://github.com/fffh1/AdaptMol. The training datasets, evaluation dataset, and the model checkpoint are available at https://huggingface.co/fffh1/AdaptMol/tree/main.

## Abbreviations

OCSR: Optical Chemical Structure Recognition
SMILES: Simplified Molecular-Input Line-Entry System
CNN: Convolutional Neural Network
RNN: Recurrent Neural Networks
LSTM: Long Short-Term Memory
ViT: Vision Transformer
MMD: Maximum Mean Discrepancy
MHSA: Multi-Head Self-Attention
FFN: Feed-Forward Network
OCR: Optical Character Recognition
GPUs: Graphics Processing Units
